# Depletion of ATP Limits Membrane Excitability of Skeletal Muscle by Increasing Both ClC1-Open Probability and Membrane Conductance

**DOI:** 10.3389/fneur.2020.00541

**Published:** 2020-06-19

**Authors:** Pieter Arnold Leermakers, Kamilla Løhde Tordrup Dybdahl, Kristian Søborg Husted, Anders Riisager, Frank Vincenzo de Paoli, Tomàs Pinós, John Vissing, Thomas Oliver Brøgger Krag, Thomas Holm Pedersen

**Affiliations:** ^1^Department of Biomedicine, Aarhus University, Aarhus, Denmark; ^2^Mitochondrial and Neuromuscular Disorders Unit, Vall d'Hebron Institut de Recerca, Universitat Autònoma de Barcelona, Barcelona, Spain; ^3^Centro de Investigación Biomédica en Red de Enfermedades Raras (CIBERER), Madrid, Spain; ^4^Department of Neurology, Rigshospitalet, Copenhagen Neuromuscular Center, University of Copenhagen, Copenhagen, Denmark

**Keywords:** skeletal muscle, ATP, membrane excitability, fatigue, membrane conductance, ClC-1, McArdle disease

## Abstract

Activation of skeletal muscle contractions require that action potentials can be excited and propagated along the muscle fibers. Recent studies have revealed that muscle fiber excitability is regulated during repeated firing of action potentials by cellular signaling systems that control the function of ion channel that determine the resting membrane conductance (*G*_*m*_). In fast-twitch muscle, prolonged firing of action potentials triggers a marked increase in *G*_*m*_, reducing muscle fiber excitability and causing action potential failure. Both ClC-1 and K_ATP_ ion channels contribute to this *G*_*m*_ rise, but the exact molecular regulation underlying their activation remains unclear. Studies in expression systems have revealed that ClC-1 is able to bind adenosine nucleotides, and that low adenosine nucleotide levels result in ClC-1 activation. In three series of experiments, this study aimed to explore whether ClC-1 is also regulated by adenosine nucleotides in native skeletal muscle fibers, and whether the adenosine nucleotide sensitivity of ClC-1 could explain the rise in *G*_*m*_ muscle fibers during prolonged action potential firing. First, whole cell patch clamping of mouse muscle fibers demonstrated that ClC-1 activation shifted in the hyperpolarized direction when clamping pipette solution contained 0 mM ATP compared with 5 mM ATP. Second, three-electrode *G*_*m*_ measurement during muscle fiber stimulation showed that glycolysis inhibition, with 2-deoxy-glucose or iodoacetate, resulted in an accelerated and rapid >400% *G*_*m*_ rise during short periods of repeated action potential firing in both fast-twitch and slow-twitch rat, and in human muscle fibers. Moreover, ClC-1 inhibition with 9-anthracenecarboxylic acid resulted in either an absence or blunted *G*_*m*_ rise during action potential firing in human muscle fibers. Third, *G*_*m*_ measurement during repeated action potential firing in muscle fibers from a murine McArdle disease model suggest that the rise in *G*_*m*_ was accelerated in a subset of fibers. Together, these results are compatible with ClC-1 function being regulated by the level of adenosine nucleotides in native tissue, and that the channel operates as a sensor of skeletal muscle metabolic state, limiting muscle excitability when energy status is low.

## Introduction

Skeletal muscle contractions support human overall health, underlying both body movement and posture, and ventilation. The ability to sustain contractile function over time, which enables prolonged physical activity during exercise, is both highly variable between different muscles and individuals, and determines the onset of muscle-fatigue (i.e., a state in which this contractile function becomes impaired and the muscular power output gradually declines). Multiple cellular mechanisms are likely to contribute to fatigue during exercise, depending on the type of exercise and type of muscle fibers being recruited by the nervous system (1).

The energy consumption of skeletal muscle increases dramatically when contracting during exercise, and a correlation between the metabolic state and performance of the muscle is well-known (2). Exhaustion of the muscular metabolic state may introduce fatigue, and excessive muscle fatigue is a well-recognized symptom in patients suffering from in-born metabolic myopathies involved in glycogen breakdown such as myophosphorylase deficiency (McArdle disease) (3). Mechanistically, several steps in the excitation-contraction coupling that link neuronal command to muscle force production are known to be sensitive to fluctuations in metabolites during exercise (2, 4).

Action potential excitation and propagation are essential steps in the normal activation of skeletal muscle fibers to generate muscle contractions, and surface membrane ion channels and ion transport proteins are key factors determining the electrophysiological properties and excitability of muscle fibers. The ClC-1 chloride (Cl^−^) ion channel is a voltage-gated chloride channel (5), that is exclusively expressed in skeletal muscle to physiological relevant levels (5–7). This ion channel, which only has a single channel conductance of about 1 pS (8), accounts for 80% of the total ion permeability of the surface membrane of resting muscle fibers, a feature that is highly conserved among muscle fiber types and species (9–13).

The equilibrium potential for Cl^−^ is close to the resting membrane potential (*V*_*m*_) in muscle fibers, because Cl^−^ is predominantly passively distributed across the muscle fiber surface membrane. Together, these properties of Cl^−^ (i.e., high Cl^−^ membrane conductance (*G*_*Cl*_), passive Cl^−^ distribution, and the equilibrium potential that is close to resting *V*_*m*_) enforce ClC-1-mediated Cl^−^ currents to stabilize the resting membrane potential. Therefore, *G*_*Cl*_ and muscle excitability are inversely related, with reduction in *G*_*Cl*_ enhancing muscle fiber excitability while a rise in *G*_*Cl*_ dampening excitability. A significant rise in *G*_*Cl*_ may even compromise muscle excitability and prevent muscle fiber activation, indicating that ClC-1 has the capability to serve as a physiological gatekeeper of the first step in the excitation-contraction coupling that underlies muscle activation (14–16).

During the last decade, the understanding of the biophysical properties of ClC-1, and the cellular signaling systems that regulate ClC-1 expression and function, has been greatly expanded. Interestingly, studies from expression systems suggest that adenosine nucleotides (i.e., ATP, ADP, and AMP) regulate the voltage dependency of the ClC-1 channel by binding to the large C-terminal part of the channel, between two cystathionine-β-synthase *(CBS)* domains (17–19). These studies show that adenosine nucleotide binding shifts the activation voltage of ClC-1 in the depolarizing direction, which implies that a loss of cellular adenosine levels could inversely cause the activation voltage of ClC-1 to shift in the hyperpolarizing direction, leading to an increased open probability at the resting membrane potential with consequent loss of muscle excitability. As ATP, ADP, and AMP had similar effects on ClC-1, the combined adenosine nucleotide levels would have to decrease for the channel to shift toward an increased opening probability. However, it is currently not clear whether ClC-1 is also regulated by adenosine nucleotides in native tissue.

Support for ClC-1 as a metabolic sensor in native tissue came from observations in action potential firing muscle fibers, where it was reported that prolonged action potential firing resulted in substantial ClC-1 activation in rodent fast-twitch muscle, leading to reduced muscle fiber excitability in combination with action potential excitation- and propagation failure. As this ClC-1 activation was rapidly reversible, this activation likely reflects physiological relevant ClC-1 regulation during skeletal muscle activity (20). Although ClC-1 is present in, and regulates the membrane conductance of, both fast- and slow-twitch fiber-types, this substantial action potential firing-induced ClC-1 activation was only observed in fast-twitch muscle fibers. As fast-twitch muscle fibers have been shown to undergo larger ATP-depletion during exercise than slow-twitch fibers (21), these results are compatible with a possible role of ClC-1 as a metabolic sensor linking metabolic state to muscle fiber excitability.

The cellular signaling that underlies ClC-1 activation during prolonged action potential firing remains incompletely understood, and there is no data available describing the relationship between adenosine nucleotides and ClC-1 activation in native skeletal muscle tissue. The present study aimed to explore if depletion of adenosine nucleotides results in increased ClC-1 opening in native tissue of muscle fibers. Such a mechanism of ClC-1 regulation may underlie the ClC-1 activation during prolonged action potential firing that has been observed in fast-twitch muscle fibers of rat and mice (20, 22, 23). Given that this ClC-1 activation has been observed in muscles with a fast-twitch fiber-type only, the present study also explored whether similar rises in membrane conductance can be triggered in both rat muscle with a slow-twitch fiber-type and in human abdominal muscle with a mixed fiber-type under conditions of compromised glycolysis. Moreover, as a proof-of-principle, this study explored whether skeletal muscle fibers of McArdle mice were prone to an accelerated increase in membrane conductance during repetitive action potential firing.

## Materials and Methods

### Animals and Ethical Approval

C57BL/6 mice (3–4 week old, male/female), Wistar rats (12–14 week old, females), and McArdle mice (12 months old, male/female) were housed under 12 h light/dark conditions at 21°C and fed *ad libitum*. Wistar rats and C57BL/6 mice were euthanized by CO_2_ exposure/inhalation, and McArdle mice were anesthetized with s.c. injection of Hypnorm/Midazolam (0.01 ml/g, 25% Hypnorm, 25% Midazolam, 50% H_2_O) and subsequently euthanized by cervical dislocation. Extensor digitorum longus (EDL), flexor digitorum brevis (FDB), or soleus (SOL) muscles were freshly harvested for experimental procedures. Both EDL and FDB contain predominantly fast-twitch fibers, while SOL contains predominantly slow-twitch fibers (24–26).

McArdle (PYGM^R50X/R50X^) and representative wild-type (PYGM^wt/wt^) mice were generated on a C57BL/6 background and extensively characterized as previously described (27, 28).

All handling and use of animals complied with Danish Animal Welfare regulations and was conducted in accordance with the European Convention for the Protection of Vertebrate Animals used for Experimental and Other Scientific Purposes (ETS 123). Experimental procedures were approved by the animal welfare officer at Aarhus University or Copenhagen University, and the complied with Danish Animal Experiments Inspectorate (permit no 2014-15-0201-00041).

### Human Muscle Fibers and Ethical Approval

Human rectus abdominis muscle (HAM) biopsies were isolated as described elsewhere (29). In short, bundles of well-defined muscle fibers with tendinous insertions at both ends, measuring approx. 8 cm × 2 cm × 1, cm were isolated from human subjects undergoing for abdominal surgery. Subjects had no known neuromuscular diseases. HAM contains an almost equal contribution of fast- and slow-twitch fibers (30). The isolation and use of human skeletal muscle was approved by the Danish Ethics Committee, Region Midtjylland, Comité I (reference number 1-10-72-20-13), and experiments were performed in accordance with the Declaration of Helsinki. Informed consent was obtained from test subjects.

### Whole Cell Patch Clamping

#### Muscle Preparation

C57BL/6 FDB muscles were incubated in HEPES-Ringer solution [122 mM NaCl, 15 mM Na-HEPES, 9 mM HCl, 2.8 mM KCl, 1.27 mM CaCl_2_, 1.2 mM MgSO_4_, 1.2 mM KH_2_PO_4_, 5 mM D-glucose and collagenase type 1 (2 mg/ml)] for 45 min at 37°C. Collagenase was removed by a serial removal of supernatant followed by washing in solution without collagenase. To dissociate the individual fibers, the muscles were triturated with Pasteur pipettes of decreasing pore size, and only fibers with maximal length of 400 μm and clear striation were used for experiments. Fibers were stored in HEPES-Ringer solution at 5°C, and all electrophysiological recordings were performed within 10 h after dissection.

#### Experimental Setup

The dissociated FDB fibers were mounted in an organ bath with a constant perfusion of extracellular (EC) solution [72.5 mM TEA-Cl, 1.2 mM CaCl_2_, 6 mM MgSO_4_, 120 mM HEPES, 20 μM nifedepine, pH 7.3 by CsOH (around 40 mM)], and they were allowed to adhere to the glass surface for 5 at least minutes before perfusion of chamber was started. Glass patch pipettes, with a maximum resistance of 2.5 MΩ, were pulled using a micropipette puller model P-97 (Sutter Instruments, California, US), and were back-filled with either an intracellular (IC) solution containing 0 mM ATP [10 mM BAPTA, 180 mM HEPES, 1.3 mM MgSO_4_, 5 mM Na_2_SO_4_, 15 mM CsCl, pH 7.2 by CsOH (around 95 mM)] or 5 mM ATP [10 mM BAPTA, 174.5 mM HEPES, 5 mM Na_2_ATP, 5.85 mM MgSO_4_, 15 mM CsCl, pH 7.2 by CsOH (around 90 mM)].

To achieve similar free Mg^2+^ content in both solutions, the required amount of MgSO_4_ was calculated with Maxchelator (Chris Patton, Stanford University, US). Muscle contractions were prevented by BAPTA (a highly selective Ca^2+^ chelator) ensuring a proper seal during recordings. Moreover, the composition of the IC and EC solutions prevented any K^+^ (0 mM K^+^, TEA-Cl and Cs^+^) and Ca^2+^ (nifedepine) currents and allowed only negligible Na^+^ currents (10 mM Na^+^ in the IC solution).

#### Electrophysiology

ClC-1 current amplitude was determined in individual muscle fibers using the whole-cell voltage clamp technique controlled by a MultiClamp 700B Amplifier (Molecular Devices, California, US) and sampled using an analog-to-digital Micro1401-3 converter (Cambridge Electronic Design, UK). The voltage protocol and data acquisition were controlled using Signal 6.03 software (Cambridge Electronic Design, UK) with a sampling frequency of 40 kHz.

**Figure 1:**
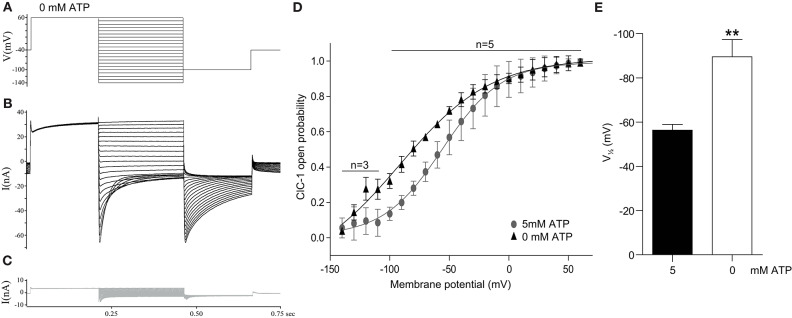
ATP depletion results in an increased ClC-1 open probability. Isolated murine fast-twitch FDB muscle fibers were subjected to whole-cell patch clamping. **(A)** The protocol used to evoke currents through ClC-1 in fibers exposed to pipette solution with 0 or 5 mM ATP. **(B,C)** Examples of whole-cell current traces from a fiber before **(B)** and after **(C)** incubation with 400 μM 9-AC. **(D)** The average open probability of ClC-1 with either 0 or 5 mM ATP in the pipette. Data are depicted as average ± SD and the solid line represents a sigmoidal fit to the data. **(E)** The average $V_{½}$ for fibers exposed to pipette solution with 0 or 5 mM ATP. A *T*-test was performed to test significance of observed differences between 0 mM ATP (*n* = 5) and 5 mM ATP (*n* = 5) and *p* < 0.01** is depicted.

The glass pipettes were inserted in the experimental bath under a slight positive pressure. At first contact between the pipette and the fiber membrane, the positive pressure was released, and a negative pressure was generated to establish a tight seal between the glass pipette and the membrane. A minimum resistance of 1.5 GΩ was obtained before going whole-cell. To secure proper equilibration of the IC solution, no recordings were made for the first 8 min after establishing whole cell voltage control. Experiments were only possible in fibers where contractions were abolished by the BAPTA that was diffused into the muscle fibers. Hence only fibers where dialysis of the intracellular space had taken place could be used. During the recordings, the serial resistance was compensated to at least 90%. The voltage protocol started at −40 mV (holding potential), and consisted of multiple cycles that each contained a 200-ms depolarization step (+60 mV), a 250-ms variable “test” voltage step (between +60 to −140 mV), and a 200-ms constant step (−100 mV) (Figure 1A) from which the instantaneous current or tail current was obtained. This is a similar procedure to what has been used elsewhere (31). The holding potential of −40 mV corresponds to the calculated equilibrium potential for Cl^−^ under the experimental conditions and this meant that ClC-1 current would be minimal at the holding potential. Some leak current did develop over the course of the experiments that at least in part could reflect incomplete ClC-1 deactivation between sweeps. This was corrected for in the data analysis. The extracellular Cl^−^ concentration, which is lower relative to more physiological levels (74.9 vs. ~140 mM), was chosen to minimize ClC-1 current to improve voltage control during the voltage steps. The variable “test” voltage started at −140 mV in the first cycle and increased with 10 mV with each cycle. Three repeats were performed at each test voltage. In a few fibers, the test voltage started at −100 mV but this did not appear to affect the average data and it was judged acceptable to include all data. Measurements were performed first in absence and subsequent in presence of 400 μM 9-anthracenecarboxylic acid (9-AC). Leak current was subtracted in all recordings, and having recordings from the same fibers before and after 9-AC meant that residual leak-current and capacitive artifact could be eliminated by subtracting the measurement with 9-AC from the measurement without 9-AC. The 9-AC sensitive current was taken to represent ClC-1 current.

The open probability (*P*_*o*_) of ClC-1 at the different clamping voltages was estimated from the peak tail currents at the −100 mV constant voltage step that followed the variable test voltage steps (Figures 1A–C). By comparing the peak tail currents that followed each test voltage step to the tail current obtained when the test pulse was +60 mV, the open probability (*P*_*o*_) could be calculated and plotted against the clamping voltage step. Based on these calculations, the data from each fiber was fitted to the 4-parameter sigmoidal function (Equation 1):

(1) $$\begin{array}{l} P_{o\left(V\right)}=P_{o\left(r\right)}+\frac{a}{1+\exp\left(\frac{V_{½}-V}{b}\right)} \end{array}$$

Where *P*_*o*(*V*)_ is the channel open probability at the voltage *V, P*_*o*(*r*)_ is a constant that represents the residual channel opening at infinitely hyperpolarized conditions, *a* represents the amplitude from the residual current to the maximal ClC-1 current at +60 mV (maximal *P*_*o*_), *b* represents the slope, and $V_{½}$ represents the midpoint potential at which 50 % of *a* is achieved.

### Two- and Three-Electrode Membrane Conductance Measurements in Action Potential Firing Muscle Fibers

#### Experimental Setup

Freshly excised skeletal muscles from Wistar rat (EDL or SOL), mice (McArdle mouse EDL), or human (HAM) were used in these experiments. In all cases, the muscles were mounted under slight tension in an organ bath containing Krebs-Ringer solution (122 mM NaCl, 25 mM NaHCO_3_, 2.8 mM KCl, 1.2 mM KH_2_PO_4_, 1.2 mM MgSO_4_, 1.3 mM CaCl_2_). The solution was supplemented with 5.0 mM D-glucose (Sigma Aldrich, Denmark) (CTRL), 5.0 mM 2-deoxy-D-glucose (2DG, Sigma Aldrich, Denmark)/100 IU insulin (Humulin R, Lilly, USA), or 5.0 mM D-glucose + 100 μM iodoacetate (IAA, Sigma Aldrich, Denmark) as indicated. For experiments with 2DG, muscles were exposed to 2DG/insulin for at least 3 h before experiments were initiated. This was done to enable the proper loading of 2DG as facilitated by insulin into the muscle fibers to impose the metabolic challenge of compromise glycolytic flux. Other muscles were incubated in the organ bath for at least 20 min prior to initiating experiments. Pharmacological inhibition of ClC-1 was achieved by supplementing HAM muscle with 100 μM 9-Anthracenecarboxylic acid (9-AC, Sigma Aldrich, Denmark). 10 nM tetrodotoxin (TTX, Tocris, UK) was used to prevent spontaneous action potential firing in the presence of 9-AC. The buffer was continuously gassed with a mixture of 95% O_2_/5% CO_2_ (pH ≈ 7.4) and kept at 30°C. Glass pipettes (8–12 MΩ) were pulled using a micropipette puller model P-97 and back-filled with 2 M potassium-citrate.

To enable electrodes to remain inserted in the muscle fibers while repeatedly firing action potentials, the contractile activity of the muscles was reduced by 50 μM N-benzyl-p-toluene sulphonamide (BTS, Toronto Research Chemicals, Canada) for EDL (32), and 25 μM blebbistatin (Sigma Aldrich, Denmark) for SOL or HAM. Previous studies have shown that these compounds have minimal effect on muscle excitability (20) and BTS was shown to reduce energy consummation during activity only by 20% (33). BTS and blebbistatin were dissolved in DMSO, which resulted in a maximal DMSO concentration of 0.15%, which did not affect resting conditions in muscles. BTS and blebbistatin were added to the solution that perfused the experimental chamber at least 30 min prior to start of measurements, and reduction in contractile activity was visually verified.

Individual muscle fibers and the electrodes were visualized using a Nikon DS-Vi1/Nikon Eclipse FN1 microscope. Electrodes were connected to TEC-05X clamp amplifier systems (NPI, Germany), and to a Power1401 converter (Cambridge Electronic Design, UK). The voltage protocol and data acquisition were controlled using Signal software (Cambridge Electronic Design, UK) with a sampling frequency of >30 kHz.

#### Electrophysiology

Electrophysiology was performed using the two-electrode technique for McArdle mice and three-electrode technique for Wistar rats and human muscle biopsy material as described previously (23).

##### *G*_*m*_ determination using three electrodes

**Figure 2:**
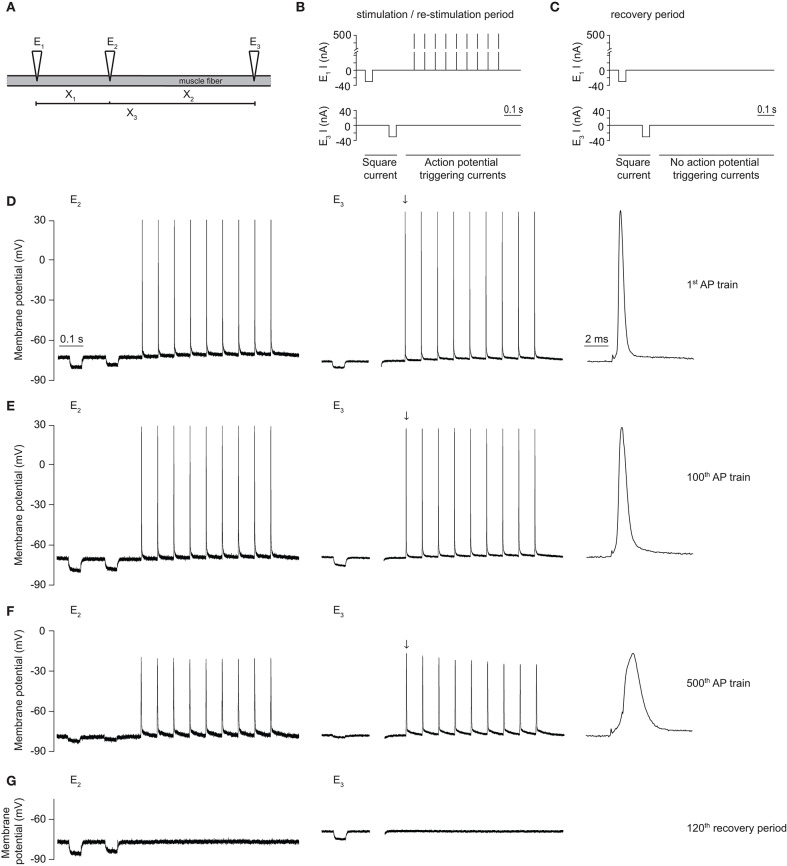
Voltage protocol and representative traces of a rat EDL-muscle control fiber using the three-electrode technique. **(A)** Schematic depiction of the placement of three electrodes (E_1_, E_2_, E_3_) in a muscle fiber, in order to obtain three inter-electrode distances (X_1_, X_2_, X_3_). **(B)** Schematic depiction of the current injections through E_1_ and E_3_ during the *G*_*m*_ determination and action potential stimulation protocol. The used protocol, with a total duration of 1000 ms, consisted of two square hyperpolarizing currents used for determination of *G*_*m*_ (−30 nA, 50 ms duration) and a train of large amplitude depolarizing current pulses to trigger a short train of action potentials [9 pulses (500 nA, 15 Hz, 1 ms)]. **(C)** The recovery protocol was similar to **(B)** except that the action potential firing pulses were not included. **(D–G)** Representative traces of the membrane potential recorded with E_2_ and E_3_ in a rat EDL muscle fiber during a CTRL experiment, with the first action potential measured with E_3_ (black downward arrow) depicted at higher time resolution on the right side. **(D)** Traces obtained during injection of the first action potential (AP) train of the protocol, **(E)** during the 100th action potential train of the protocol, and **(F)** during the 500th action potential train of the protocol. **(G)** The last (120th) trace of the recovery protocol.

Three electrodes (E_1_-E_3_) were placed into the same fiber, where the inter-electrode distance between E_2_-E_3_ (X_1_) was twice the inter-electrode distance between E_1_-E_2_ (X_2_, Figure 2A). Current *(I)* was first, injected by E_1_ while the steady membrane potential response (Δ*V*) was measured with E_2_ and E_3_, and subsequently, current was injected by E_3_ while Δ*V* was measured with E_1_ and E_2_. This resulted in the measurement of Δ*V* at three different distances on the fiber (X_1_-X_3_). The transfer resistances at the different inter-electrode distances were calculated by dividing Δ*V* by *I*, and they were plotted against the respective inter-electrode distances and, finally fitted to the cable equation (Equation 2) that applies to cells of infinite length with a membrane represented by a parallel RC circuit (34). *R*_*in*_ and λ refer to the fiber input resistance and length constant of the fiber that were obtained from the cable equation fit (Equation 2):

(2) $$\begin{array}{l} \Delta V\left(x\right)=\Delta V_{x=0}\exp\left(-x\lambda^{-1}\right)=IR_{in}\exp\left(-x\lambda^{-1}\right) \end{array}$$

Δ*V(x)* represents the steady state change in membrane potential at position *x* relative to where the current was injected at *x* = *0*. From *R*_*in*_ and λ the membrane resistance per unit length of fiber (*r*_*m*_), the intracellular resistance per length of muscle fiber (*r*_*i*_), and the membrane conductance per unit length of fiber (*g*_*m*_) were calculated:

(3) $$\begin{array}{l} r_{m}=2R_{in}\lambda ={\left(g_{m}\right)}^{-1} \end{array}$$

(4) $$\begin{array}{l} r_{i}=\frac{2R_{in}}{\lambda } \end{array}$$

*G*_*m*_, the surface membrane specific conductance, was then calculated by dividing *g*_*m*_ with the surface area per unit length of fiber (*FSA*). In resting muscle fibers a common approach for estimating *FSA* is to first determine the cross sectional area of the fiber (*CSA*) from *r*_*i*_ assuming a constant value for the specific sarcoplasmic resistivity (*R*_*i*_), here taken to be 180 Ω cm as determined in resting fibers (35), using Equation 5:

(5) $$\begin{array}{l} r_{i}=\frac{R_{i}}{CSA} \end{array}$$

Next, by assuming the muscle fiber to be a perfect cylinder, *FSA* can be calculated from *CSA* as:

(6) $$\begin{array}{l} FSA=\sqrt{4\pi CSA} \end{array}$$

and *G*_*m*_ can be then be calculated as:

(7) $$\begin{array}{l} G_{m}=\frac{g_{m}}{FSA}=\frac{g_{m}}{\sqrt{4\pi CSA}} \end{array}$$

##### Stimulation protocol mimicking muscle activity using three-electrode technique

Repeated firing of action potentials with interspaced *G*_*m*_ determinations was performed in muscle fibers from Wistar rat (SOL and EDL) and human (HAM) muscles with the three-electrode technique. Three electrodes were inserted in these muscle fibers, as described above under *G*_*m*_ *determination*, and current was injected according to a current-injection protocol with a pre-defined number of cycles. Each cycle of the protocol started with two square negative currents (−30 nA, 50 ms) which were injected through E_1_ and E_3_, respectively (used for the *G*_*m*_ *determination* as described above), followed by either a train of action-potential generating positive currents injected through E_1_ (i.e., stimulation protocol) or a rest period for the remainder of the cycle (i.e., recovery protocol). All three electrodes recorded the membrane potential at their respective locations.

For Wistar rat SOL/EDL fibers, the stimulation protocol [1000 ms, 9 pulses (500 nA, 15 Hz, 1 ms)] was repeated 500x to trigger 4500 action potentials in control fibers, or 200x to trigger 1800 action potentials in 2DG-exposed fibers. Subsequently, the recovery protocol [1000 ms, 0 pulses] was repeated 120x during which no action potentials were triggered and only *G*_*m*_ was monitored, which was followed by a re-stimulation period in which the stimulation protocol was again repeated 120x to trigger 1080 action potentials. The stimulation duration used in rat muscle fibers was based on a previous publication, which describes the presence and absence of a rise in *G*_*m*_ in EDL and SOL in this time-frame, respectively (20).

For HAM fibers, the stimulation protocol [1400 ms, 10 pulses (300 nA, 30 Hz, 1 ms)] was repeated 143x to trigger 1430 action potentials in all conditions. The protocol was ceased before it reached 1430 action potentials when failure in action potential excitation/propagation was observed. The stimulation duration used in HAM fibers was based on the development of the rise in *G*_*m*_ under glycolysis inhibition and the protocol is similar to a previous publication (15).

##### Stimulation protocol mimicking muscle activity using two-electrode technique

The two-electrode technique was chosen for experiments with EDL muscles from McArdle mice as this approach has a higher success rate, and these animals were in very short supply. Likewise, the stimulation protocol was chosen to maximize the success rate of the experiments. Two electrodes (E_1_-E_2_) were placed in close proximity (≈0 μm) within one fiber, of which E_1_ injected current according to a current-injection protocol with a pre-defined number of cycles, and E_2_ measured the membrane potential at *x* = *0*. Each cycle of the protocol started with a square negative current (−30 nA, 50 ms), which was used to measure the fiber *R*_*in*_. This was followed either by a train of action-potential generating positive currents (i.e. stimulation protocol) or a rest period for the remainder of the cycle (i.e., recovery protocol).

The stimulation protocol [1500 ms, 15 pulses (300 nA, 30 Hz, 1 ms)] was repeated 200x to trigger 3000 action potentials. Subsequently, the recovery protocol [1500 ms, 0 pulses] was repeated 100x during which no action potentials were triggered and only *R*_*in*_ was monitored, which was followed by a re-stimulation period in which the stimulation protocol was again repeated 100x to trigger 1500 action potentials. The stimulation was continued until the *R*_*in*_ started to decline drastically reflecting the development of the rise in *G*_*m*_.

These experiments thus measured *R*_*in*_ and to convert this into *G*_*m*_ during activity, the three electrode techniques described above in 2.4.2.2. was used to determine *G*_*m*_ in inactive fibers of the same muscle. *G*_*m*_ during muscle activity could then be estimated using equation 8 as described previously (22).

(8) $$\begin{array}{l} G_{m\left(t=x\right)}=G_{m\left(t=0\right)}\frac{{R_{in\left(t=0\right)}}^{2}}{{R_{in\left(t=x\right)}}^{2}} \end{array}$$

##### Fitting properties to determine the timing and size of the rise in G_*m*_

Based on observations of *G*_*m*_ during muscle activity the data from each fiber was fitted to the following 4-parameter sigmoidal function as previously described, to determine the timing and maximal size of the rise in *G*_*m*_ (22) (Equation 9):

(9) $$\begin{array}{l} G_{m}=G_{m_{\left(\min\right)}}+\frac{\Delta G_{m_{\left(\max\right)}}}{1+\exp\left(\frac{t_{x}-t}{b}\right)} \end{array}$$

Where *G*_*m*_ represents the *G*_*m*_ at the time *t, G*_*m*(*min*)_ represents the minimal *G*_*m*_, Δ*G*_*m*(*max*)_ represents the difference between the minimal and maximal *G*_*m*_, *b* represents the slope, and *t*_*x*_ represents the time at which *G*_*m*_ is 50% of Δ*G*_*m*(*max*)_.

### Statistics and Data Handling

All fibers in which the resting membrane potential recorded by one of the electrodes depolarized beyond −60 mV during the experimental procedure were excluded from analyses. The number of analyzed fibers (n) is depicted for each group in the graph legends, and originated from at least 3 different animals per group for all experiments performed on C57BL/6 mice and Wistar rats. Data from McArdle mice and representative controls were shown per individual mouse, and human biopsy data originated from one HAM muscle. Data was presented as mean ± SEM unless otherwise indicated. Differences between data depicted in two groups were tested with Student's *t*-test, and differences between data depicted in multiple groups were tested with ANOVA and Bonferroni's *post-hoc* test (parametric distributions) or Kruskal-Wallis and Dunn's *post-hoc* test (non-parametric distributions) where appropriate. Differences between binary data depicted in multiple groups were tested using the Fisher's exact test, and corrected for multiple comparisons. *p*<0.05 was considered significant, and *p*<0.05^*^, *p*<0.01^**^, *p*<0.001^***^ are depicted in the figures.

## Results

### ATP-Dependent ClC-1 Open Probability in Native Skeletal Muscle Fibers

To study whether ClC-1 opening is sensitive to differences in intracellular ATP levels ([ATP]) in native skeletal muscle fibers, whole cell patch clamping was performed on isolated fast-twitch muscle fibers from mice with either 0 or 5 mM ATP in the pipette solution. The protocol for determining ClC-1 current in the muscle fibers and the calculation of the voltage dependent open probability (*P*_*o*_) of ClC-1 was as performed as previously described elsewhere (31) (Figure 1A). Ionic currents were measured before (Figure 1B) and after addition of 400 μM 9-AC (Figure 1C) in the same muscle fiber, and the ClC-1-specific ionic current was calculated by subtracting the ionic current obtained with 9-AC from the current obtained without 9-AC. In general, the membrane current in the presence of 9-AC was markedly reduced, showing that ClC-1 facilitated the majority of the current flow under these experiment conditions (compare black and gray traces in Figures 1B,C). There was no significant difference in peak ClC-1 current at +60 mV between groups exposed to 5 and 0 mM ATP pipette solutions (5 mM ATP: −40 ±13 nA, 0 mM ATP: −34 ±16 nA, *p* = 0.553). Also, there was no difference in currents between the groups after 9-AC exposure.

The calculated *P*_*o*_ at each specific membrane potential was plotted against that membrane potential, and subsequently fitted to the sigmoidal function in Equation 1 (Figure 1D). The parameters from the fits of individual fibers were obtained and comparison of parameters was performed between groups. The slope (5 mM ATP: 33 ±23 mV, 0 mM ATP: 33 ±8 mV, *p* = 0.944) and residual opening probability at the most hyperpolarized potentials (5 mM ATP: −0.074 ± 0.232, 0 mM ATP: −0.181 ± 0.319, *p* = 0.563) were not different between the groups. The membrane voltage that resulted in 50% of the voltage dependent ClC-1 current to be activated, $V_{½}$, was shifted significantly in the hyperpolarizing direction in fibers subjected to a pipette solution with 0 mM ATP (Figures 1D,E).

### A Repetitive Action Potential Firing-Induced Rise in *G_*m*_* Occurs Only in Fast- and Not in Slow-Twitch Fibers Under Conditions With Functional Glycolysis

The initial series of experiments in muscle fibers was conducted to verify previous findings that repetitive action potential firing resulted in large increases in *G*_*m*_ with subsequent loss of muscle excitability in fast-twitch (EDL) but not in slow-twitch (SOL) muscle fibers from rat. Experimentally, the three-electrode technique (Figure 2A) was used and a 1000 ms stimulation protocol was repeated 500 times in each fiber. During each run of the protocol, *G*_*m*_ was first determined with two square currents, and, subsequently, 9 action potentials were triggered in the fiber (Figure 2B). After 500 runs of the protocol, the action potential firing was ceased, and the recovery of *G*_*m*_ was monitored during 120 runs of the protocol without triggering action potentials (Figure 2C). Finally, action potential triggering was re-started and 120 runs of the action potential triggering protocol (Figure 2B) were performed.

**Figure 3:**
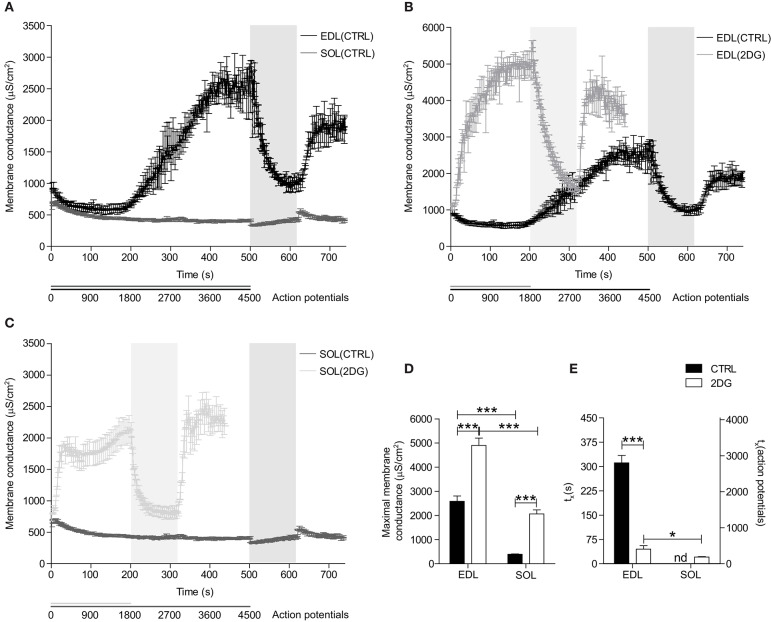
Changes in *G*_*m*_ during repetitive action potential firing in fast- and slow-twitch fibers under control conditions and with inhibition of glycolysis. **(A)** *G*_*m*_ of EDL (black line) and SOL (gray line) muscle fibers in buffer with 5.0 mM D-glucose is depicted over time. *G*_*m*_ of EDL **(B)** and SOL **(C)** muscle fibers in solution with 5.0 mM D-glucose (dark line) or 5.0 mM 2DG + 100 IU insulin (light-gray line) is depicted over time. *G*_*m*(*max*)_ **(D)** and time until 50% of *G*_*m*(*max*)_ is reached (*t*_*x*_) **(E)** were extracted from sigmoid-fitted data and depicted. CTRL fibers were subjected to 500x the stimulation protocol [1000 ms, 9 pulses (500 nA, 15 Hz, 1 ms)], 120x the recovery protocol [1000 ms, 0 pulses], and to a re-stimulation period that consisted of 120x the stimulation protocol. 2DG fibers were subjected to 200x the stimulation protocol, 120x the recovery protocol, and to a re-stimulation period that consisted of 120x the stimulation protocol. Data from EDL-CTRL and SOL-CTRL fibers depicted in panel **(A)** is also depicted as CTRL data in panel **(B,C)**, respectively. Gray vertical bars represent the recovery period during the stimulation protocol. ANOVA with Bonferroni's *post-hoc* tests were performed to test significance of observed differences between EDL-CTRL (*n* = 4), EDL-2DG (*n* = 12), SOL-CTRL (*n* = 18), and SOL-2DG (*n* = 32), and *p* < 0.05* or *p* < 0.001*** are depicted.

In line with previous work from our group, *G*_*m*_ showed a biphasic response during repetitive action potential stimulation in EDL but not in SOL muscle fibers (15, 20, 22). Thus, *G*_*m*_ decreased during the first 1500 action potentials (phase 1) of repetitive action potential firing in both EDL and SOL muscle fibers (Figures 2E, 3A). This has previously been shown to primarily reflect protein kinase C-mediated inhibition of ClC-1 ion channels in both muscle fiber types (15, 20), and was not the aim of the current manuscript. In EDL fibers only, *G*_*m*_ rapidly increased after around 1500–2000 action potentials (phase 2) (Figures 2F, 3A). *G*_*m*_ returned to baseline values during the recovery period, and subsequently increased quickly upon re-stimulation (Figures 2G, 3A). During prolonged action potential firing, the action potential amplitude, and depolarization and repolarization speed decreased gradually over time (Figures 2D–F).

### The Repetitive Action Potential Firing-Induced Rise in *G_*m*_* Occurs in Both Fast- and Slow-Twitch Fibers Under Conditions of Compromised Glycolysis

To test if the regulation of *G*_*m*_ is dependent on a functional ATP supply, pharmacological inhibition of glycolysis (i.e., by replacing 5.0 mM D-glucose with 5.0 mM 2DG + 100 IU insulin) was performed in the above-described experiments, but with a shorter action potential stimulation period (200 s, 1800 action potentials). The stimulation period was shorter in these experiments because less action potentials were needed to trigger the activity-induced rise in *G*_*m*_. Glycolysis-inhibited EDL muscle fibers showed an accelerated phase 2-like increase in *G*_*m*_ directly after the onset of action potential-firing (Figures 3B,E). Moreover, this rise in *G*_*m*_ was steeper and reached a higher maximal *G*_*m*_ before becoming stable compared to muscle with fully functional glycolysis (Figures 3B,D). Consequently, an initial phase 1-like decrease in *G*_*m*_ was not observed in glycolysis-inhibited EDL muscle fibers. Similar to control conditions, *G*_*m*_ returned to baseline values in the recovery period, and subsequently increased quickly upon re-stimulation (Figure 3B).

Similar to the results from glycolysis-inhibited EDL muscle fibers, inhibition of glycolysis resulted in a rapid phase 2-like increase in *G*_*m*_ in SOL muscle fibers (Figures 3C,E). Moreover, the increase in *G*_*m*_ in glycolysis-inhibited SOL muscle fibers was also steep but plateaued earlier (±450 action potentials), and at a much lower maximal *G*_*m*_ compared with the maximal *G*_*m*_ of glycolysis-inhibited EDL muscle fibers (Figures 3B–D). Like in glycolysis-inhibited EDL muscle fibers, the *G*_*m*_ of glycolysis-inhibited SOL muscle fibers returned to baseline values in the recovery period, and subsequently increased quickly upon re-stimulation (Figure 3C).

### Inhibition of Glycolysis Triggers an Action Potential Firing-Induced ClC-1-Dependent Rise in *G_*m*_* in Human Muscle Fibers

**Figure 4:**
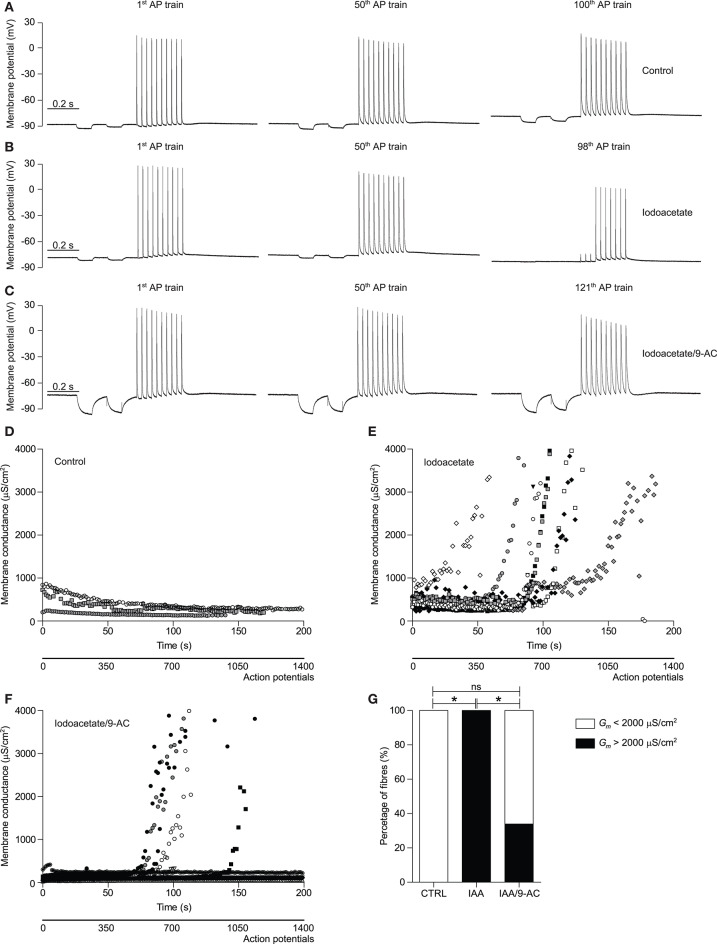
Glycolysis-inhibition resulted in a repetitive action potential firing-induced ClC-1-dependent rise in *G*_*m*_ in human muscle fibers. **(A–C)** Representative traces of the membrane potential recorded with E_2_ (for schematic depiction of electrodes see Figure 2A) of a control (CTRL) **(A)**, 100 μM iodoacetate (IAA) subjected **(B)**, and 100 μM IAA + 100 μM 9-AC + 10 nM Tetrodotoxin subjected **(C)** human HAM muscle fiber during the 1st, 50th, and ~100th action potential (AP) train of the stimulation protocol have been depicted. The protocol, with a total duration of 1400 ms, consisted of two square hyperpolarizing currents used for determination of *G*_*m*_ (−30 nA, 50 ms duration) and a train of large amplitude depolarizing current pulses to trigger a short train of action potentials [10 pulses (300 nA, 30 Hz, 1 ms)]. **(D–F)** *G*_*m*_ of CTRL **(D)**, IAA subjected **(E)**, and IAA/9-AC subjected **(F)** individual HAM muscle fiber are depicted over time. **(G)** The percentage of HAM muscle fibers that showed an increase in *G*_*m*_ that was >2000 μS/cm^2^ during the first 200 s of action potential stimulation. Fisher's exact tests corrected for multiple comparisons were performed to test significance of observed differences between CTRL (*n* = 3), IAA (*n* = 9), and IAA/9-AC (*n* = 12), and *p* < 0.05* is depicted.

Previous experiments, using a similar approach to determine *G*_*m*_ changes during repetitive action potential firing, have shown that although ClC-1 inhibition during the first phase of activity is also present in human muscle, a prolonged activity-induced rise in *G*_*m*_ has not been observed in the first 2000 action potentials (15). To determine whether this rise in *G*_*m*_ can, similar to SOL muscles from rat, be brought out with inhibition of glycolysis, a series of experiments with the three-electrode technique was conducted in HAM muscle in the absence and presence of iodoacetate (IAA). Indeed, no repetitive action potential firing-induced rise in *G*_*m*_ was observed in control conditions in human muscle fibers during the 200 s of running the stimulation protocol (Figures 4A,D,G). Similar to the results from glycolysis-inhibited rat SOL muscle fibers, however, inhibition of glycolysis resulted in the appearance of a phase 2-like increase in *G*_*m*_ in all tested human muscle fibers (Figures 4B,E,G). This increase in *G*_*m*_correlated strongly with action potential-excitation failure, as can been clearly seen in the action potential train from the representative trace depicted in the third panel of Figure 4B. To study the relative contribution of ClC-1 to the observed increase in *G*_*m*_, the above described glycolysis-inhibition experiments were repeated in presence of the ClC-1 inhibitor 9-AC. ClC-1 inhibition prevented the rise in *G*_*m*_ in 75% of the measured glycolysis-inhibited human muscle fibers (Figures 4C,F,G). As ClC-1 inhibition resulted in a binary effect on the rise of *G*_*m*_, measured fibers were depicted individually and not as mean values (Figures 4D–F), and the percentage of fibers that showed an increase in *G*_*m*_ during the first 200 s of the stimulation protocol were depicted (Figure 4G). During prolonged action potential firing, the action potential amplitude, and depolarization and repolarization speed decreased gradually over time. Moreover, 9-AC-mediated inhibition of ClC-1 currents resulted in a decreased repolarization speed at baseline (Figures 4A–C). Since the access to human muscle was limited, IAA was used to inhibit glycolysis, as this approach was much faster and allowed paired measurements to be performed in the same preparations. This was not possible with 2DG that required substantial pre-incubation.

### Action Potential Firing-Induced Rise in *G_*m*_* Is Accelerated in Murine McArdle EDL Muscle Fibers

**Figure 5:**
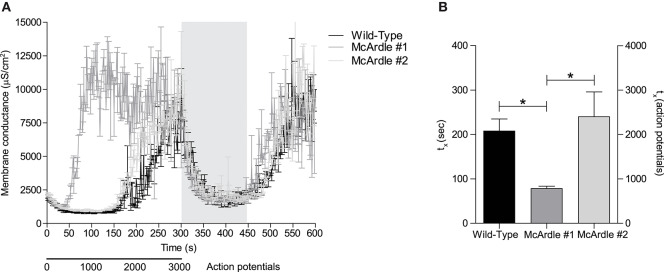
Rise in *G*_*m*_ is accelerated in murine McArdle EDL muscle fibers compared with wild-type EDL muscle fibers. **(A)** Murine EDL muscle fibers of McArdle (gray lines) and genetic wild-type (black line) mice were subjected repetitive action potential stimulation and *G*_*m*_ of is depicted over time. **(B)** Time until 50% of *G*_*m*(*max*)_ is reached was extracted from sigmoid-fitted data and depicted. Fibers were subjected to 200x the stimulation protocol [1500 ms, 15 pulses (300 nA, 30 Hz, 1 ms)], 100x the recovery protocol [1500 ms, 0 pulses], and to a re-stimulation period that consisted out of 100x the stimulation protocol. Gray vertical bars represent the recovery period during the stimulation protocol. Kruskal-Wallis and Dunn's *post-hoc* test was performed to test significance of observed differences between wild-type (*n* = 9), McArdle #1 (*n* = 9), and McArdle #2 (*n* = 13), and *p* < 0.05* is depicted.

The above-described experiments in rat and human muscles showed that *G*_*m*_ rise can be markedly accelerated by pharmacological inhibition of glycolysis. This is compatible with ClC-1 being a metabolic sensor that can switch open and compromise muscle excitability if the metabolic state of muscle fibers becomes critical. To further explore for a link between membrane conductance and glycolysis, a genetic model for impaired muscle glucose and energy homeostasis (i.e., McArdle mouse model) was used. Interestingly, the observations from the two analyzed McArdle mice showed marked differences regarding the onset of the repetitive action potential firing-induced rise in *G*_*m*_ (Figure 5A). Experimentally, the two-electrode technique was used and a 1500 ms stimulation protocol was repeated 200 times (300 sec, 3000 action potentials), followed by a recovery period (150 s, 0 action potentials), and a re-stimulation period in which the same stimulation protocol was repeated 100 times (150 s, 1,500 action potentials) in each fiber. The muscle fibers from the first McArdle mice showed a consistently accelerated increase in *G*_*m*_ compared with wild-type muscle fibers. However, the muscle fibers from the second McArdle mouse did not show an accelerated increase in *G*_*m*_ compared with wild-type (Figures 5A,B). Both McArdle and wild-type muscle fibers showed a similar return of *G*_*m*_ to baseline during the recovery period as well as a similar rapid increase in *G*_*m*_ upon re-stimulation (Figure 5A). Taken together, the combined observations are compatible with malfunctional glycolysis (pharmacologically or genetically induced) being able to introduce an accelerated rise in *G*_*m*_ during muscle activity. Given the limited amount of data available from these McArdle mice, this data must be considered with appropriate reservations, and more experiments are required to explore this in further detail.

## Discussion

The metabolic requirement of skeletal muscle fibers increases many-fold during physical activity. Although the muscular ATP replenishing capacity is extensive, a fiber type-specific exhaustion of the metabolic state occurs during intense exercise. Indeed, [ATP] was reported to decline by 80% in fast-twitch and 25% in slow-twitch human muscle fibers after 25 s of maximal exercise. These changes in [ATP] were generally accompanied by corresponding increases in inositol monophosphate (IMP) levels, showing that the exercise led marked decline in the adenosine nucleotides pool in the active muscle fibers (21). The amplitude of this decrease suggest that severe energy depletion is a physiologically relevant event during high-intensity activity, especially for fast-twitch muscle fibers.

The physiological importance of a decline in adenosine nucleotides and altered metabolic profile for muscle function has been studied extensively and several cellular mechanisms involved in the excitation-contraction coupling are sensitive to the specific metabolites. A correlation between metabolic state of muscle fibers and their performance is evident, suggesting that metabolically driven fatigue mechanisms exist in skeletal muscle. The current study shows that depletion of adenosine nucleotides results in an increased ClC-1 open probability in skeletal muscle, and that the inhibition of glycolysis results in an accelerated increase in membrane conductance during repetitive action potential firing in both slow- and fast-twitch muscle fiber types. Interestingly, this accelerated increase of membrane conductance was present in both rodent and human muscle fibers, and it was found to be ClC-1 dependent. Moreover, as a proof-of-principle, this study indicates that fast-twitch muscle fibers from McArdle mice, that have an impaired intra-muscular glucose homeostasis, exhibit accelerated increase of membrane conductance during repetitive action potential firing. Together, these results suggest that ClC-1 function is regulated by the level of adenosine nucleotides, and that the channel operates as a sensor of the metabolic state in skeletal muscle fibers. ClC-1 thereby represents a mechanistic link between metabolism and muscle fiber excitability that can be envisioned to have a role in muscle fatigue during exercise and, possibly, neuromuscular disease including McArdle disease.

### Whole Cell Patch Clamp of Muscle Fibers Show Increased ClC-1 Opening Probability Under Conditions of Low Intracellular ATP Levels

Previous patch clamp studies using heterologous expressed ClC-1 reported that adenosine nucleotides cause a pH dependent inhibition of ClC-1 (17, 36, 37). Others were, however, unable to reproduce these findings (38). This apparent discrepancy between studies was later explained by the high sensitivity of the ATP-ClC-1 binding to the redox state of the cell (39). Although voltage clamping of mammalian muscle fibers has already been used to measure chloride currents for over 25 years (40), the adenosine nucleotide sensitivity of ClC-1 in native mammalian muscle fibers has not been explored. We now show that ATP-depletion results in a hyperpolarizing shift of the ClC-1 open probability in native skeletal muscle fibers, confirming the expression system-obtained data from previous studies in a more physiological relevant setting (17, 36).

The whole cell patch clamping used in the current study does have some experimental limitations that must be considered. As muscle fibers have a complex cellular geometry (size and composition), complete voltage control during the experiments may have been compromised. At least it must be assumed that the T-tubular membrane was not fully under voltage control due to the T-tubular lumen imposing substantial resistance to current flow (16). Furthermore, it is unlikely that the level of ATP was fully controlled by the pipette solution due to the large fiber volume (41). The change in the ClC-1 activation with 0 mM ATP in the pipette was nevertheless of a clear magnitude, and the limitations considered here indicate that our results may be an underestimation of the effect of ATP on ClC-1.

The ATP depletion-induced shift in activation of ClC-1 results in a higher ClC-1 opening at resting membrane potential in native muscle fibers and, predictably, would cause an increase in the resting membrane conductance of resting skeletal muscle fibers. Adenosine nucleotide mediated regulation of ClC-1 could therefore provide a mechanistic link between metabolic state of muscle fibers and their excitability. This was next explored in muscle fibers during repetitive firing of action potentials.

### Effect of Pharmacological Inhibition of Glycolysis on Regulation of Resting Membrane Conductance During Repetitive Action Potential Firing in Muscle Fibers of Rat and Human

Previous studies showed that repetitive firing of action potentials results in the activation of both ClC-1 and K_ATP_ ion channels, which markedly increase the resting membrane conductance leading to compromised muscle fiber excitability (22). This increase in membrane conductance has thus far only been observed in fast-twitch muscle fibers and not in slow-twitch fibers. This fiber-type specific activation of ClC-1 and K_ATP_ ion channels is compatible with fast-twitch fibers undergoing the largest depression in adenosine nucleotides during intense muscle activity. To further explore the role of the metabolic state for the activity-induced rise in membrane conductance, two series of experiments were conducted using both rat and human muscles to confirm translatability from rodent to man.

First, the effect of pharmacological inhibition of glycolysis with 2DG on the regulation of the resting membrane conductance during repeated firing of action potentials was explored in rat fast- and slow-twitch muscle fibers. With glycolysis inhibition, the rise in membrane conductance happened rapidly after onset of action potential firing in both fast- and slow-twitch muscle fibers. This rise in membrane conductance was observed significantly later in untreated fast-twitch fibers, and it was not observed at all in untreated slow-twitch fibers. This shows that the ion channels that trigger the increased membrane conductance during prolonged action potential firing have the capacity to be activated in both types of muscle fibers. The absence of the increase in membrane conductance in slow-twitch fibers under control conditions is likely due to the relatively stable metabolic state in active slow-twitch fibers, where adenosine nucleotide levels might not decrease to a level that triggers ClC-1 activation.

Second, when similar experiments were conducted in untreated abdominal rectus muscle fibers from human, the onset of muscle activity caused a reduction in the membrane conductance as has been reported previously (15). This state of low membrane conductance during onset of action potential was previously shown to be caused by protein kinase C-mediated ClC-1 inhibition, and has been associated with well-preserved muscle fiber excitability (15). In our experiments, the low membrane conductance and well-preserved excitability was maintained for the complete protocol of action potential firing in the untreated human muscle fibers. When similar experiments were conducted in the presence of iodoacetate, a marked and consistent rise in membrane conductance was observed during the repeated action potential firing in the human muscle fibers. This rise in membrane conductance led to complete loss of muscle fiber excitability in many fibers and, importantly, this activity-induced rise in membrane conductance was significantly abolished in the presence of a ClC-1 inhibitor. Although these findings show that ClC-1 has the capacity to activate during metabolic compromised conditions in human muscle fibers, it remains to be established if ClC-1 also activates under control conditions if stimulations are either intensified or extended beyond 2000 action potentials in these fibers. It is possible that such ClC-1 activation during prolonged muscle activity would occur in a subset of human fibers most closely comparable to the EDL muscle fibers of the rat.

Taken together, the findings from these two series of experiments show that the rise in membrane conductance during repeated firing of action potentials is a conserved capacity between different muscle fibers and species. In the currently explored rat slow-twitch and human muscle fibers, the rise in conductance was only observed with glycolytic inhibition. Although this is an indication that these muscle fibers were not metabolically challenged by the imposed activity, and that the adenosine nucleotide levels were well-preserved in these muscle fibers during the activity, especially for human abdominal muscle fibers longer stimulation periods are needed to verify this. Although we did not study intra-muscular adenosine nucleotide concentrations in the current study, it is known from literature that 2DG and iodoacetate both result in depletion of adenosine nucleotides in challenged cardiac muscle (42). Nevertheless, it would be interesting to study if the rise in membrane conductance starts at similar intra-muscular adenosine nucleotide concentrations in glycolysis-inhibited and control fibers and possibly make inter-species comparisons.

### Role of ClC-1 in the Rise in Membrane Conductance During Repeated Action Potential Firing

Collectively, the patch clamping data and the observations in human muscle during repeated firing of action potentials suggest that the ClC-1-mediated chloride conductance is largely responsible for the accelerated rise in membrane conductance during prolonged muscle activity. The primary data implicating ClC-1 channels in the rise in membrane conductance during muscle activity stems, in the present experiments, from the observation from human muscle fibers where pharmacological blockage of ClC-1 resulted in the abolishment of the rise in membrane conductance. This data is in line with previous data, showing that the rise in membrane conductance during repetitive action potential firing in rat fast-twitch muscle was to a large extend mediated by ClC-1 activation (22). Although we clearly show an important role for ClC-1 for the rise in membrane conductance during prolonged action potential firing, a possible role for K_ATP_ should not be disregarded. Since both ClC-1 and K_ATP_ channels are sensitive to the metabolic state of muscle fibers, these channels may be speculated to introduce some degree of redundancy in the capacity of muscle fibers to shut down muscle excitability in the case of challenged metabolic state (22).

Although the membrane conductance is extremely important for functional excitation-contraction coupling, multiple cellular mechanisms that regulate down-stream sarcoplasmic reticulum Ca^2+^ release are sensitive to the metabolic state of muscle fibers. Indeed, previous studies not only showed that the sarcoplasmic reticulum Ca^2+^ release was greatly impaired by both low [ATP] and by the inhibition of glycolysis with 2DG or IAA, but they also showed that the opening of the skeletal muscle Ca^2+^ release channel Ryanodine-receptor 1 (RyR1) itself is dependent on ATP-binding (43, 44). Interestingly, both ClC-1 and RyR1 seem to have similar sensitivities to [ATP], where a decline below 1 mM ATP causes activation or inhibition, respectively. Therefore, it is possible that adenosine nucleotides have a universal role as regulators of the excitation contraction coupling and that coordinated shutdown of muscle activation by several cellular mechanisms can take place if [ATP] declines below 1 mM.

### Effect of Genetically Compromised Glycolysis on Regulation of the Resting Membrane Conductance During Repetitive Firing of Action Potentials—Role in Neuromuscular Disease

Although the currently used McArdle mice were generated to model human McArdle disease (28), they were included in this manuscript as a, proof-of-concept, genetic model for impaired muscle glucose and energy homeostasis. These mice were characterized by complete absence of the glycogen phosphorylase (a.k.a. myophosphorylase), and in a previous study they were shown to have a dramatically decreased time to fatigue (28). Moreover, the currently included EDL muscle of McArdle mice showed clear signs of structural damage, and displayed impaired twitch and tetanic force production (45). The currently presented data, that shows that the membrane conductance rises after around 1500 action potentials in McArdle wild-type (i.e., C57BL/6 PYGM^wt/wt^) muscle fibers, corresponds exactly to reliable membrane conductance data from an extensive previous characterization of C57BL/6 EDL fiber cable parameters (23). From the two different McArdle knock-in mice (PYGM^R50X/R50X^) tested here, only one showed a clear accelerated rise in membrane conductance. Although it is unclear why this response was observed in only one of the mice, it is possible that not all animals were affected equally by the mutation. As all the reported data was obtained in fibers that remained polarized throughout the experimental protocol, and the membrane conductance of all fibers returned quickly to baseline during the recovery period, all fibers were considered healthy and viable and, thus, the data to be reliable. The currently presented McArdle data only served as proof-of-concept, to verify the pharmacological glycolysis inhibition data in a non-pharmacological genetic system. Together with the data from pharmacological glycolysis inhibition, these data converge to a conclusion that the observed acceleration of the rise in membrane conductance is physiologically relevant and a conserved response to low energy status. Due to the limited amount of observations that we gathered from McArdle mice, substantial precautions must be taken when extrapolating and interpreting the current data the McArdle disease *per se*.

Combined, our data clearly indicates the importance of a functional energy homeostasis for proper membrane conductance physiology and the data shows that ClC-1 acts as a metabolic sensor that increases membrane conductance and limits muscle excitability when energy status is low.

## Data Availability Statement

The datasets generated for this study are available on request to the corresponding author.

## Ethics Statement

The animal study was reviewed and approved by Danish Animal Experiments Inspectorate. The studies involving human participants were reviewed and approved by Danish Ethics Committee, Region Midtjylland, Comité I. The patients/participants provided their written informed consent to participate in this study.

## Author Contributions

PL, FP, TP, JV, TK, and THP contributed conception and design of the study. PL, KD, KH, and AR contributed to acquisition of data. PL, KH, AR, TK, and THP contributed to analysis or interpretation of data. PL and THP contributed to writing the first draft of the manuscript. KH and AR wrote sections of the manuscript. All authors contributed to manuscript revision, read and approved the submitted version.

## Conflict of Interest

The authors declare that the research was conducted in the absence of any commercial or financial relationships that could be construed as a potential conflict of interest.
